# Counseling for Health: How Psychological Distance Influences Continuance Intention towards Mobile Medical Consultation

**DOI:** 10.3390/ijerph20031718

**Published:** 2023-01-17

**Authors:** Fuyong Lu, Xintao Wang, Xian Huang

**Affiliations:** 1 Institute of Education and Economy Research, University of International Business and Economics, No. 10, Huixindongjie, Chaoyang District, Beijing 100029, China; 2 School of Business, Renmin University of China, No. 59, Zhongguancundajie, Haidian District, Beijing 100872, China

**Keywords:** mobile medical consultation, continuance intention, information systems continuance model, psychological distance theory, pandemic-induced anxiety

## Abstract

As mobile healthcare services entered the public sight with high frequency during the COVID-19 pandemic, patients are increasingly recognizing the effectiveness of mobile medical consultation (MMC). Earlier studies have investigated what influences continuance intention (CI) towards MMC, but few studies have scrutinized it from the perspective of patients’ psychological distance. We formulated a framework to examine the psychological factors influencing CI towards MMC by integrating the information systems continuance model and psychological distance theory. The framework was validated using the partial least squares structural equation modeling (PLS-SEM) approach and data from 475 MMC users in China. The empirical results revealed that immediacy, telepresence, intimacy, and substitutability were significant predictors of CI, while satisfaction mediated these pathways. Pandemic-induced anxiety positively moderated the effect of immediacy on satisfaction and the effect of satisfaction on CI. Practical implementations for MMC healthcare practitioners, designers, and marketers are drawn.

## 1. Introduction

At the beginning of 2020, the outbreak of COVID-19 forced healthcare institutions, especially in developing countries, into a state of overload and dramatically reduced available per capita healthcare resources [1]. An adequate medical resource, such as disease diagnosis, medical prevention, and scientific treatment, is closely related to physical health, mental vitality, and life quality. Hence, as an effective way to access medical resources, mobile medical consultation (MMC) has become a global trend [2]. MMC addresses the growing demand for non-contact medical consultations, which contributed to consumer psychological identification with online healthcare [3]. According to iResearch [4], the scale of online healthcare users in China has reached 298 million, with an annual growth rate of 38.7%. The four leading health apps, Ping An Good Doctor, So Young, Chunyu Yisheng, and Haodf, were used by almost 18.9 million monthly active consumers in 2022 [5]. To properly understand the effect of communication between MMC and users, we explored the psychological factors that influence users’ continuance intention towards MMC, which are the basis for forming a harmonious communication of MMC in society.

Continuance intention (CI) generally refers to user post-adoption intention after the initial adoption of technology. It has been a popular theme in MMC within the last five years, which is perceived as a typical means of measuring MMC success. However, previous studies on CI towards MMC have been conducted mainly from a technical perspective [6,7], combined with particular unidimensional psychological features such as trust [8,9], health anxiety [10], and justice perceptions [11]. Few studies investigated CI towards MMC from a comprehensive psychological view. Given that the COVID-19 pandemic has plagued the world for three years, the foothold of the healthcare system is extending from technology to people [12]. Investigating CI towards MMC from a psychological perspective is necessary because a common state is revealed when users face MMC: as time goes by, the frequency of use decreases among online patients. For example, Baumel et al. [13] revealed that user retention of healthcare devices dropped dramatically after one month of installation. Specifically, Fleming et al. [14] detailed that 80% of all participants in medical interventions logged in to the service less than twice. These figures illustrate that a significant number of patients discontinue MMC after initial adoption. Psychological factors, other than technical characteristics, may explain why users continue to use or abandon MMC.

Therefore, to explain the CI towards MMC, we extended the information systems continuance model by integrating four psychological agents (immediacy, telepresence, intimacy, and substitutability) based on psychological distance theory [15]. Scholars have introduced psychological distance theory into the information system domain to explain CI towards online commerce [16], virtual environments [17], and artificial intelligence [8]. Several studies on mobile health systems have shown that psychological mechanisms (e.g., mental health [18], IT identity [19]) have a non-negligible impact on the exploration of continuance intentions. However, psychological distance theory has not been applied to predict the continuance intentions of healthcare technologies, especially MMC. According to psychological distance theory, psychological distance is the subjective experience of something being near or far from us in the here and now, which fills our brain, influences our emotions, and guides our choices and actions [15]. We expect that MMC users can perceive a lower psychological distance and focus on what is beneficial to their health when interacting with mobile doctors. Gaining users’ CI requires continually lowering their psychological distance and meeting their expectations for MMC, which is a challenge for mobile healthcare services.

Pandemic-induced anxiety describes the degree to which pandemic concerns are incorporated into an individual’s daily activities [20]. During the COVID-19 pandemic, previously accustomed living and working conditions change, which may cause anxiety and panic in societal groups [21,22,23]. To mitigate the distress and psychological impact of the pandemic, patients often proactively seek professional medical help from mobile healthcare services and try to adapt to them without physically engaging in the patient–physician interaction [24]. Therefore, we hypothesized that patients who implemented adaptive measures against external uncertainty induced by pandemic anxiety were more likely to adopt mobile technology-dependent medical consultations consistently. To date, pandemic-induced anxiety has not been examined in the context of MMC, so this gap provides a novel aspect to our study. Thus, this paper contributes to the theory by integrating psychological distance factors (immediacy, telepresence, intimacy, and substitutability) as well as pandemic-induced anxiety into the information systems continuance model.

The purposes of this study were, firstly, to determine the psychological mechanisms explaining CI towards MMC and to validate the proposed theoretical model with empirical data from Chinese MMC users. Secondly, this study examined the moderating effect of pandemic anxiety on CI towards MMC. This study considered the psychological antecedents of CI in the context of China; thus, our findings contribute to the existing literature from an Eastern perspective. The remainder of this paper is structured as follows: the following section presents the theoretical basis of this study and reviews psychological research on MMC users. The methodology describes the overview of research design, measurement development, data collection and sample, and data analysis methods. The results are then presented. Finally, we discuss implications to the literature and theory, as well as positing discussions, limitations, and future research agendas.

## 2. Theoretical Framework

### 2.1. Information Systems Continuance Model (ISCM)

Our theoretical framework begins with the ISCM. The ISCM was developed based on expectation confirmation theory [25] and the technology acceptance model [26], which attempts to explain the continued use of technology after initial adoption. Continuation intention is one of the core concepts that researchers need to focus on when exploring the factors that influence individuals’ acceptance of information technology, and it is also the most central component of the information systems continuance model. In this model, continuation intention describes the intention of individuals to continue using information systems, and satisfaction is one of the most important determinants of CI for technologies that have been adopted [27,28]. The ISCM is extensively employed in individuals’ CI of technologies such as e-learning technologies [29], bike-sharing apps [30], mobile wallet apps [31], online banking [32], and social networking technologies [33,34]. Furthermore, in the context of healthcare technologies, existing studies have examined the positive relationship between satisfaction and CI and highlighted it as a key factor motivating people to repurchase the same services [6,35,36,37,38]. Therefore, the ISCM, a proven technology adoption model, provides the necessary theoretical framework for our study to explore the continuance intention towards mobile medical consultation. Based on the ISCM, our study examines the mechanisms that psychological distance serves as an antecedent to satisfaction and CI in the MMC context.

### 2.2. Psychological Distance Theory (PDT)

According to PDT, psychological distance reflects the degree of difference between an individual’s mental and real activity [15]. A greater difference means a greater psychological distance and vice versa. Psychological distance can be subdivided into four dimensions: temporal, spatial, social, and hypothetical [15]. These four dimensions are defined as follows: temporal distance refers to the psychological distance between now and earlier or later; spatial distance refers to the psychological distance from here to nearer or farther; social distance refers to the psychological distance from self to familiar or unfamiliar; and hypothetical distance refers to the probability of determining that something will happen [39]. PDT has been applied in studies related to information systems [16,40,41]. However, most of the existing literature based on PDT treated psychological distance as a single-dimensional predictor and did not distinguish its sub-categories associated with specific information systems (e.g., MMC). There is also an absence of exploring which specific features of psychological distance are beneficial in facilitating users’ assessment of CI, especially in the MMC setting.

Since MMC has reached the diffusion phase of development [42], it is thus essential to determine which psychological distance can be classified in light of the characteristics of MMC. On the basis of relevant findings, the psychological distance in this study is divided into four cardinal features (immediacy, telepresence, intimacy, and substitutability) that assist us in distinguishing MMC from OMC (offline medical consultation). First, immediacy and telepresence are highly recognized qualities of psychological distance and are broadly identified in existing research [43,44,45]. The above two features are crucial contributors to the superiority of mobile scenarios over offline environments. Second, intimacy is another psychological property of mobile services. The lower the intimacy between individuals, the greater the social distance between individuals in MMC [46]. Increasing group intimacy is one of the reasons why users desire to use mobile services [47,48]. Third, substitutability is regarded as the fourth characteristic of MMC because medical consultations act as a sense of substitutability that is produced by perpetuating competition among MMC and OMC [49]. As mentioned previously, the following four psychological distances are proposed in the context of MMC: immediacy, telepresence, intimacy, and substitutability. Table 1 presents the definitions of each construct.

## 3. Hypothesis Development

### 3.1. Immediacy (IM)

IM refers to the degree of time delay in communication or feedback between multiple subjects [50]. It captures the temporal psychological distance people develop between something happening and perceiving it [44,55]. Therefore, in this study, immediacy is defined as the ability of MMC to respond rapidly to users’ consulting needs. Examination of multiple mobile technologies demonstrated the significant impact of IM on CI. Prior studies are in favor of a direct effect of IM on CI in MMC [56].

Most users decide to seek medical advice from MMC for a time-saving purpose [57]; thus, it is important that the MMC is considered immediate and able to feedback with real-time information and foster satisfaction [58]. Moreover, several studies in mobile healthcare suggested that the effect of IM on CI is fully or partially mediated by satisfaction [59]. Thus, users who are satisfied with MMC are more likely to continue using MMC. Therefore, we propose the following:

**Hypothesis** **1 (H1).** *IM has a positive influence on satisfaction.*

**Hypothesis** **1a (H1a).** *The influence of IM on CI is mediated by satisfaction.*

### 3.2. Telepresence (TP)

TP measures the realism of a virtual environment in which users can feel the sensation of “physical contact” [45,51]. The significant effect of TP on user behavior intention has been supported by studies concerning information systems technology [60,61]. Considering TP as a form of “being there” in mobile services, Lim et al. [62] found that TP is correlated with CI. Furthermore, An et al. [63] found that, in virtual tourism, the effect of TP on behavioral intention was partially mediated by satisfaction.

Given the resemblance between virtual tourism and MMC (high human–computer interaction), we anticipate the impact of TP on satisfaction to parallel prior research [64]. With mobile technology, users expect to describe their condition as if physically in front of physicians or to immerse themselves in patient–physician interaction through video and wearable devices wherever they are. Eliminating spatial distance is a practical advantage offered by MMC through IS technology and telemedicine technology, adding to user satisfaction. Thus, TP of MMC significantly influences user satisfaction, which is a prerequisite mechanism for CI generation. Therefore, we propose the following:

**Hypothesis** **2 (H2).** *TP has a positive influence on satisfaction.*

**Hypothesis** **2a (H2a).** *The influence of TP on CI is mediated by satisfaction.*

### 3.3. Intimacy (IN)

IN describes feelings of closeness and emotional connection, including intense interpersonal liking, external moral support, and a combined ability to tolerate the shortcomings of important others [65]. The positive connection between IN and CI is supported by research in web-based services, running apps, and mobile apps [66]. Empirical studies in mobile apps [66], micro-blogging services [67], and business-to-business [68] also found that the relationship between IN and CI was mediated by satisfaction.

However, the effect of IN on CI has not been proven in healthcare technology (especially MMC). According to PDT, IN affects an individual’s psychological state and thus motivates the acceptance of mobile health [69]. As an informal communication instrument, MMC offers a way through which people can foster interpersonal connections, such as sharing medical news with strangers or acquaintances. Interpersonal factors (e.g., self-disclosure) can influence individual satisfaction [70,71]. Therefore, we hypothesized that patients’ IN in MMC influences their psychological perceptions, enhancing satisfaction and thus stimulating CI. Therefore, we propose the following:

**Hypothesis** **3 (H3).** *IN has a positive influence on satisfaction.*

**Hypothesis** **3 (H3a).** *The influence of IN on CI is mediated by satisfaction.*

### 3.4. Substitutability (SU)

Hendee and Burdge [72] reported that SU refers to the interchangeability of activities that meet the needs, motivations, and preferences of the individuals involved. In this study, SU suggests that the original offline medical behavior is not easily accessible in the real world due to the pandemic and could be replaced by alternative behavior if medical consultation activities are initiated or continued. Previous research investigated the influence of SU on users’ intention in online media [73], video platforms, mobile applications [74], and virtual reality [75] but ignored the scope of MMC and the importance of satisfaction.

Specifically, in the context of MMC, high-quality feedback, practical guidance, and a reasonable balance between offline medical consultations and alternative online behavior are important triggers for SU [76]. Healthcare feedback and disease-directed consultation are features embedded into most MMCs so that the user’s medical consultation needs can be met in accordance with their clinical condition, essentially. Thus, using MMC to attain health goals is a viable alternative to OMC [49,77]. Therefore, we hypothesized that MMC users can develop a state of perceived SU after initial use, which would enhance their satisfaction and propensity for CI. Therefore, we propose the following:

**Hypothesis** **4 (H4).** *SU has a positive influence on satisfaction.*

**Hypothesis** **4 (H4a).** *The influence of SU on CI is mediated by satisfaction.*

### 3.5. Satisfaction (SA)

SA refers to an individual’s overall evaluation of product performance to date [53]. SA (or dissatisfaction) arises from comparing individual expectations and the product’s performance [25]. SA occurs when the performance of a product exceeds an individual’s expectations; however, dissatisfaction occurs when performance falls below expectations. Thus, SA can be shaped by making comparisons in terms of expectations. Statistically significant positive effects of SA on CI have been demonstrated in several studies in healthcare technology contexts, such as clinical information systems, electronic medical records, online health communities [78], and medical consultation platforms [6,11,79]. Thus, previous studies have shown that SA can significantly predict CI in MMC. Therefore, we hypothesized that patients would be more inclined to continue using MMC if they perceive SA with the mobile health services. Therefore, we propose the following:

**Hypothesis** **5 (H5).** *SA has a positive influence on CI.*

### 3.6. Pandemic-Induced Anxiety (PA)

PA, as a negative affective reaction towards a pandemic, refers to individuals’ apprehensive feelings when they face the choice to fight against the pandemic, and it has been identified as a determinant of health attitudes and intentions [54]. Researchers proposed that individuals use mobile health as a coping mechanism to regain the sense of control lost due to PA [80].

The activities offered by mobile health are more likely to appeal to individuals with higher PA [81]. These individuals also have a greater likelihood of seeking medical information and adopting methods that can improve or maintain their health, and they are more likely to document their physical condition and share their experiences of keeping healthy with other patients or acquaintances [82]. Furthermore, mobile health services offer more options for people to improve or maintain their physical and mental health [83]. In addition, people with higher PA have better healthcare awareness and clearer health goals, so they have better judgments of satisfaction with mobile health [84]. Thus, the effects of IM, telepresence, IN, and SU on SA and the effect of SA on CI may be amplified by PA. Therefore, we propose the following:

**Hypothesis** **6 (H6).** *PA increases the predictive power of IM (H6a), TP (H6b), IN (H6c), and SU (H6d) on SA, as well as increasing the predictive power of SA on CI (H6e).*

**Figure 1:**
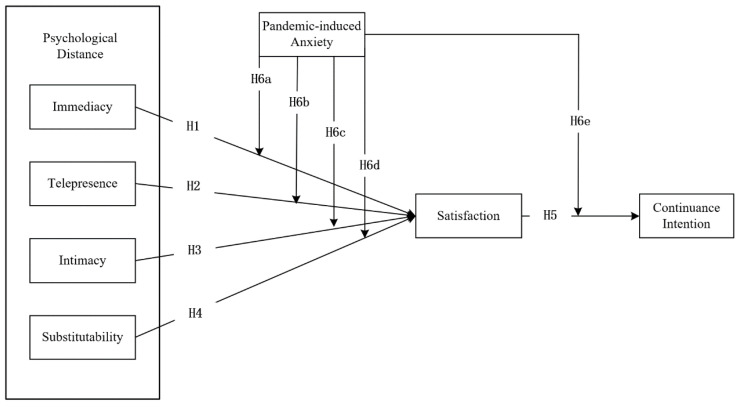
Conceptual model of the determinants of CI towards MMC.

All hypotheses for the base model are summarized in Figure 1.

## 4. Methodology

### 4.1. Overview of Research Design

This study developed a psychological distance measure to assess the relationship between psychological distance and satisfaction and between satisfaction and continuance intention. We also identified the role of pandemic-induced anxiety in the above relationships. First, focus groups were organized to determine the dimensions of psychological distance that participants considered necessary. A survey reflecting psychological distance dimensions was constructed using items from existing scales. Confirmatory factor analysis was applied to items related to psychological distance, satisfaction, continuance intention, and pandemic-induced anxiety. Then, using partial least squares structural equation modeling (PLS-SEM), a model tested the extent to which psychological distance explained MMC satisfaction, the extent to which MMC satisfaction explained continuance intention, and the moderating effect of pandemic-induced anxiety on the above mechanisms. Finally, we tested the explanatory power of psychological distance on consumer consultation satisfaction, the explanatory power of consumer consultation satisfaction on continuance intention among Chinese MMC consumers, and the moderating effect of epidemic anxiety on the above mechanisms using partial least squares structural equation modeling (PLS-SEM).

### 4.2. Measurement Development

We developed items for the study following the three-stage instrument development process (i.e., item creation, scale development, and instrument testing) suggested by Moore and Benbasat [85]. For the item creation phase, existing measurement items from previous studies were reviewed and modified to fit the mobile medical consultation setting. The scale was given to an expert group (*n* = 3) who were experienced psychologists to ensure that each item was understandable and relevant to the topic. To measure IM, we used four items adapted from Zhang et al. [50] and Okazaki and Mendez [55]. TP was measured with four items adapted from Sun et al. [60], while IN was measured with four items from Park and Lee [86] and Lin et al. [66]. We measured SU with four items adapted from Li [74]. For PA, we employed five items from Wang et al. [54] and Goyal et al. [87]. SA was measured with four items from Bhattacherjee [27]. CI was measured with four items from Bhattacherjee [27]. Items and sources of the scales are presented in Appendix A, Table A1.

For the scale development phase, a group of experts reviewed the instrument to identify ambiguous items created in the first phase. To improve the items readability, a pilot test was conducted with 25 subjects. Based on feedback from the pilot test, some measurement items were modified to ensure that the measurements were straightforward and understandable. For the instrument testing phase, another small-scale pretest was conducted with 35 subjects before data were collected for field testing. The instrument’s reliability, construct validity, and discriminant validity were examined to ensure its applicability. The psychological distance scale (PDS) from Zheng et al. [88] was chosen to assess scale validity because it was previously established to assess the same construct based on psychological distance theory.

### 4.3. Data Collection and Sample

The questionnaire was distributed through the Chinese online survey platform, Wenjuanxing (https://www.wjx.cn/ (accessed on 2 March 2022)), due to the isolation during the pandemic. This study recruited people who had a mobile medical consultation experience since 2019. The authors of this study who were trained investigators sent a web link with questions to mobile medical consultation consumers. Initially, the questionnaire was sent to 50 respondents who completed it as required and received an immediate reward (approximately USD 1). Before the questionnaire was formally sent to the public, potential respondents were asked to report whether or not they have experience with mobile medical consultation and confirm the name of the mobile medical app they recently used. If the answers were eligible, the formal phase of the questionnaire was opened. Otherwise, respondents’ access to the questionnaire was closed. We excluded respondents who completed the survey in less than one minute and submitted it repeatedly.

We used G*Power software to calculate the minimum sample size for this study. According to the study of Franque et al. [89], we set up the significance level (α) as 0.05 (i.e., 95% of confidence level), effect size (i.e., an average of the correlation coefficient) as 0.416, and power as 100% to generate that the minimum sample size for the study is 120. Research data were collected in April 2022. A total of 515 valid responses to the questionnaire were received. After examining the data, the questionnaire ultimately yielded a sample of 475 valid answers, with a validity rate of 92.2%. The most popular age group was 31–40 (39.5%). More males (53.1%) joined this study than females (46.9%), and 56.2% of participants held an Undergraduate degree. The most popular category of monthly household income was USD 1200 and 1700 (29.4%). The top year of mobile medical consultation was 1–2 years (47.2%). The most frequent mobile medical consultations were often (55.2%). Table 2 illustrates the demographic characteristics of the respondents.

### 4.4. Data Analysis Methods

We used structural equation modeling (SEM) to analyze the effects of various psychological distance factors on consumer satisfaction and continuation intentions. The SEM approach was preferred because it allows researchers to program relationships between multiple independent and dependent variables together [90]. SEM applies to this study as a mediating mechanism exists in our model. In addition, the partial least squares (PLS) method was used to test our model since PLS is well-placed for testing complex structural models because it eliminates two problems: unacceptable solutions and factorial uncertainty [91]. In addition, PLS employs a component-based model estimation method, which does not require large samples and residual distributions [92].

To summarize, we used partial least squares structural equation modeling (PLS-SEM) to examine the measurement models and structural models. There are several reasons for choosing PLS-SEM analysis over other analysis techniques. First, PLS-SEM is considered most appropriate when the study emphasizes identifying possible relationships between structures rather than the magnitude of these relationships [93]. Second, PLS-SEM is often used to avoid excluded solutions and factor uncertainty. Third, PLS-SEM is robust, ensures convergence, and reduces statistical identification problems [90]. The computational software, SmartPLS, accompanies the method used in this paper, and its version number is v.3.3.3. SmartPLS v.3.3.3 is capable of handling complex model analyses without strict data assumptions such as residual distributions and large samples. SmartPLS also can simplify procedures for modeling reflection structures while reporting the composite reliability (CR) and the average variance extracted (AVE) of validity.

## 5. Results

### 5.1. Measurement Model Analysis

Measurement model analysis includes internal reliability, convergent validity, and discriminant validity analysis. First, internal reliability was obtained by assessing Cronbach’s α and composite reliability. As demonstrated in Table 3, all Cronbach α, Rho_A, and Composite reliability were well above the threshold of 0.70, thus confirming the good internal consistency of the constructs [90]. Furthermore, we tested both validities by evaluating the value of the average variance extracted (AVE), the square root of AVE, item loadings, and cross-loadings. For good discriminant validity, the ideal value of AVE should be greater than 0.5 [94], and the square root of AVE should also be greater than the correlation coefficient between the variables. In addition, if the overall psychological distance scale (IM, TP, IN, and SU) has convergent validity in the between-scale level, then constructs should relate closely to the PDS instrument. Our findings indicate that the Pearson product-moment correlation coefficients between variables are in the expected direction. Detailed measurement information is presented in Table 4.

Moreover, the cross-loading criterion was also applied to evaluate the discriminant validity [92]. It aims to assess the loadings on the constructs and confirm that it performs better in each row. Through the calculations, we found that the factor loadings that loaded well on their constructions were significantly greater than the cross-loadings that loaded poorly on the other constructions. Results, as shown in Table 5, indicate that all the constructions are loaded higher than the cross-loadings of the other constructions in the same row, which implies that good discriminant validity for all the constructs in our study.

Typically, self-reported data may be affected by common method bias (CMB). In this paper, we adopted several measures to minimize the risk of CMB. First, the questionnaires were distributed over three non-consecutive weeks, with participants’ addresses distributed across different provinces in China. This random pattern arrangement could reduce CMB. Next, we assessed CMB through a PLS model following Podsakoff et al. [94], which compared the variance of variables explained by substantive factors and method factors. The statistics indicated that the average substantive variances (0.748) were greater than the method variances (0.531), suggesting that CMB is unlikely to be a serious problem in our study (see Appendix A, Table A2).

### 5.2. Structural Model Analysis

**Figure 2:**
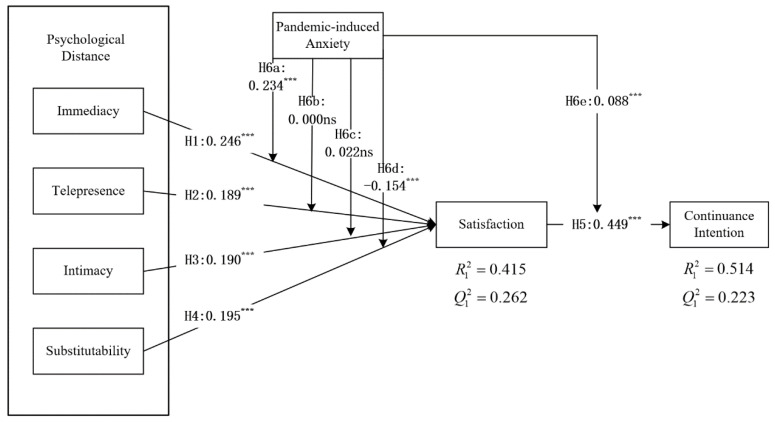
PLS result of research model testing (N = 475). Note: *** *p*-value < 0.001; ns, not significant.

In this paper, the quality of the structural model was assessed with the R-squared ($R^{2}$), effect size ($f^{2}$), and predictive correlation ($Q^{2}$). $R^{2}$ ≥ 0.25 was considered an acceptable range [90]. Acceptable $Q^{2}$ values were constrained to be equal to or greater than 0 [90]. $f^{2}$ was bounded by three theoretical intervals: 0–0.02 (small effect), 0.02–0.15 (medium effect), and 0.15–0.35 (large effect) [95]. After calculation, our model accounted for 41.5% ($R_{1}^{2}$ = 0.415) of the variance in SA and 51.4% ($R_{2}^{2}$ = 0.514) of the variance in CI. Furthermore, we applied the Stone–Geisser test and obtained $Q^{2}$ values of 0.262 and 0.223 for SA and CI, respectively, which confirmed the good predictive performance of the structural model. Then, we carried out 5000 bootstrap iterations to assess the significance of the model effects. The hypothesized paths (H1, H2, H3, and H4) were supported as IM, TP, IN, and SU all had a significant effect on SA and were in the predicted directions. More structural model results are illustrated in Figure 2 and Table 6.

### 5.3. Moderation Effects

To test the moderating effect of PA (H6a to H6e) between perceived distance characteristics (IM, TP, IN, and SU) and SA, and between SA and CI, we referred to Chin et al. [96] and Liang et al. [97], who proposed the two-stage approach for moderation analysis. Additionally, as shown in Figure 2 and Table 7, PA positively moderated the relationship of IM and SU with SA. Hence, H6a and H6e were supported. However, PA did not moderate the relationship of TP and IN with SA, which indicates that H6b and H6c were not supported. Interestingly, PA had a negative moderation role in the effect of SU on SA.

**Figure 3:**
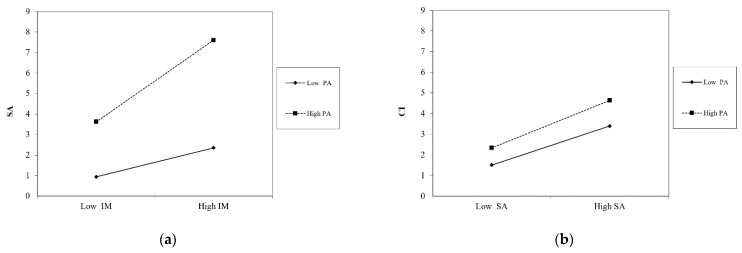
The interaction effects of PA: (**a**) the moderating effect of PA between IM and SA; (**b**) the moderating effect of PA between SA and CI.

Slope tests were performed to illustrate the results of the moderation analysis. For H6a, our analysis showed significant slopes for low PA and high PA. This finding suggests that SA increases rapidly with IM at high PA levels, while SA increases slightly with IM at low PA levels (see Figure 3a). For H6e, the low PA and high PA slopes did not significantly differ. This finding suggests that CI increases with SA regardless of the level of PA (see Figure 3b).

### 5.4. Mediation Effects

We used the bootstrapping method proposed by Zhao et al. [98] to test the mediating effect of SA. As shown in Table 8, SA mediated the relationships of IM, TP, IN, and SU with CI. The empirical results revealed that all hypothesized indirect effects were confirmed. We also tested the mediation relationship between PA and CI, but it was invalid. To obtain the magnitude of each mediating effect, we adopted the VAF indicator recommended by Helm et al. [99]. VAF represents the proportion of indirect effects to the total effect and ranges from 0 to 100%, where values above 80% mean fully mediated, values from 20% to 80% imply partially mediated, and values below 20% indicate no mediating effect [100]. In our study, the VAF values were all located between 33% and 37%, from which we can infer that SA partially moderates the effect of IM on CI. More detailed findings are presented in Table 8.

### 5.5. Control Variables

We performed significance tests for the control variables: gender (β = −0.015, *p* > 0.05), age (β = −0.006, *p* > 0.05), education (β = 0.001, *p* > 0.05), monthly household income (β = −0.022, *p* > 0.05), year of mobile medical consultation (β = −0.044, *p* > 0.05), frequency of mobile medical consultations (β = 0.014, *p* > 0.05), and found no effect of them on the study variables. After excluding all control variables, we examined the structural model, and the results indicated no differences. This reveals that the control variables did not significantly affect the path weights between the main constructs in our structural model.

## 6. Discussion

This study first developed a theoretical framework, integrating psychological distance theory and the Information systems continuance model, that aims to predict the CI toward MMC in the context of COVID-19. Our findings suggested that psychological distance (i.e., IM, TP, IN, and SU) was significantly associated with consumer satisfaction. The mediating role of consumer satisfaction between psychological distance and MMC was supported by empirical evidence. PA significantly moderated the effect of IM on SA and SA on CI.

First, the links between IM and CI and between TP and CI were mediated by satisfaction. IM and TP reflect the “ubiquity” of MMC from a temporal and spatial perspective, respectively. The more ubiquity MMC consumers feel, the higher their satisfaction will be. The result means that consumers will increase their continuance intention to some extent. IN is the essential social experience for consumers, which is the underlying philosophy for a mobile device-based system with a large user pool [101]. Especially during the COVID-19 pandemic, people preferred to use MMC technology because it helped them save time commuting and waiting in line for medical appointments, allowing them to consult medical specialists at the appropriate time and place, as they wished. The results suggested that consumers’ CI toward MMC was positively associated with satisfaction, which supports existing research on behavioral intention to adopt new health-enhancing technologies [59].

Second, the association of IN and SU with CI was mediated by satisfaction. The study pointed out that the higher the IN, the more satisfied consumers are with MMC. Our results exposed that IN may be an important factor in consumer approval of MMC. When consumers emotionally keep a low psychological distance from the technology, it can evoke an active perception of it, influencing their continuance intention towards MMC. These findings supported that affective identity with mobile health technology can further increase consumers’ intention to use the technology. In addition, the correlation between SU and CI explains why MMC technology is recommended by many organizations (e.g., government, community, and employers). Namely, MMC evolved as an alternative to offline healthcare during the pandemic by integrating online and offline healthcare functions [76]. As a result, a high acceptance of MMC was established in the community.

Third, PA positively moderated the effects of IM on SA and SA on CI. On the one hand, the relationship between IM and SA was positively moderated by PA, which indicated that consumers suffering from high levels of pandemic-induced anxiety increased their satisfaction with MMC, a finding that is broadly consistent with reality. The pandemic imposed specific social effects, such as people being forced to develop physical anxiety (e.g., insomnia) and psychological anxiety (e.g., fear), influencing consumers’ continuance intention towards MMC. On the other hand, the effect of SA on CI was positively moderated by PA, which implied that consumers suffering from high levels of pandemic-induced anxiety increased their continuance intention toward MMC. MMC contributed more to meeting consumers’ medical consultation needs when the public crisis drove people towards online medical resources. Our results confirmed that PA might exert an effect on consumers’ continuance intention towards MMC.

Fourth, the effects of TP, IN, and SU on SA were not significantly moderated by PA. First, a scarcity of medical resources during COVID-19 was the primary obstacle for patients to reach medical care rather than isolation [102]. Second, consumers may find it difficult to accurately identify emotional states when facing a public crisis due to the adverse psychological conditions caused by pandemic-induced anxiety [54]. In addition, with the stickiness of patients to offline healthcare and the limitations of MMC technology, the outbreak of the COVID-19 pandemic may not be the main concern for individuals to choose MMC as an offline healthcare alternative. These findings suggested that, when a pandemic occurs, cross-regional counseling, emotional identity, and immersive experiences may not be the primary appeals of patients toward MMC.

## 7. Conclusions

### 7.1. Implications

Considering IM, TP, IN, and SU as psychological distances, our study shows that all of these psychological distances were associated with user CI. These results yield some practical suggestions for MMC operators and sellers.

First, our study takes the lead in explaining the factors that influence the CI towards MMC from the perspective of psychological distance. MMC changed many settings of traditional medical consultation, but previous studies have not systematically investigated the psychological perceptions of individuals. We defined psychological distance in MMC and verified that it provides a mechanism for user continuance intentions. Although some studies demonstrated that mobile health systems could establish psychological mechanisms in adoption intention or continuance intention, they were still considered from a broader perspective, such as psychological well-being [18,19], and how subtle psychological distances reflect the CI towards MMC is still unclear. Thus, our study provides a theoretical basis for future MMC research. Furthermore, we adopted PA in our study, which is a new perspective on MMC. Therefore, our study contributes to the existing literature on PA in MMC.

Second, our results reveal that the image of MMC in users’ minds is directly linked to the satisfaction. Therefore, MMC operators who have not yet focused on psychological marketing may consider taking this into account to improve sales performance. For example, they can create an environment where users’ expected satisfaction and actual satisfaction are equal by showing users, in detail, the service categories before consultation, interactively communicating with users through simple and efficient screens during consultation, and collecting reviews in a rewarding manner after consultation. Sellers who have adopted psychological marketing in MMC can minimize the user’s psychological fallout during the consultation process. For example, sellers can gather more details about their services and respond quickly to users’ questions to understand user satisfaction. Some online physicians can improve their skills in guiding users. For example, online physicians may ensure comprehensive product knowledge before guiding users. Users are more likely to be firm in their CI towards MMC when online physicians provide a medical consultation experience that is as good as the offline experience.

Third, MMC operators can endeavor to improve IM, TP, IN, and SU. For example, online doctors can have an appointment function just like offline doctors, reducing the time for doctor–patient matching. The operator can also quickly help users locate their interesting consultations: for example, pushing a graphic and text report of the relevant disease for the user while they wait for the doctor’s response. Comment interaction should be more humanized rather than pure text-to-speech communication. For example, the popular emoji function in social software should be applied to the comment section of MMC, increasing users’ interactive experience. Operators can continuously enrich the functional segments of MMC to provide users with one-stop healthcare services to create unique competitiveness in mobile healthcare services.

Fourth, we suggest that operators pay attention to the plausibility of disseminating personal pandemic prevention measures. Although the relationship between PA and most psychological distances (e.g., TP, IN, and SU) in MMC is not empirically supported by the data, the results suggest that PA significantly mediates the effect of IM on CI. The temporal significance stressed by IM is considered one of the most important advantages of MMC, and this finding implies that user anxiety about the pandemic is likely to be a side factor driving the rapid development of MMC. Therefore, we propose that operators should develop their expertise in alleviating users’ pandemic anxiety, pay attention to collecting more effective demand data and service feedback, and improve users’ stickiness in the post-pandemic era.

### 7.2. Limitations and Future Research Agendas

First, the sample for this paper was Chinese respondents of MMCs. Existing research suggests that decision making on mobile healthcare system adoption varies by culture [103]. With the extensibility of our findings, future research should examine the effect of culture on CI towards MMC to measure the generalizability of our model more comprehensively.

Second, we adopted a four-dimensional structural feature for the psychological distance of MMC. Note that we chose a more established psychological theory as the basis for the model, which helps to identify the psychological distance of MMC users. Therefore, future studies can examine other variables (especially those reflecting unique medical consultation-related characteristics) as predictors in our model.

Third, because our study is based on the IS acceptance model, which models satisfaction as a single construct, we did not subdivide satisfaction into multiple criteria. However, existing research indicates that satisfaction is a multidimensional variable that includes cognitive and affective aspects [104]. Cognitive and affective satisfaction refers to consumers’ rational judgments and emotional reactions, which are currently not adequately examined in MMC. Future research could examine the impact of different psychological characteristics of MMC on multidimensional satisfaction.

## Figures and Tables

**Table 1:** Construct definitions.

Constructs	Definition	Theoretical Framework
Immediacy	Users obtain information or responses quickly and without delay in MMC (adapted from Zhang et al. [50])	Temporal (PDT)
Telepresence	Users feel like being physically transported to an offline treatment room in MMC (adapted from Zhang and Li [51])	Spatial (PDT)
Intimacy	MMC creates a strong bond and trusting relationship between patients (adapted from Chen et al. [52])	Social (PDT)
Substitutability	MMC can be a good alternative to offline medical consultations (adapted from Wu and Lu [49])	Hypothetical (PDT)
Satisfaction	Users believe MMC’s actual performance is better than expected (adapted from Johnson and Fornell [53])	ISCM
Pandemic-induced anxiety	Users’ apprehensive feelings when they face the choice to fight against the pandemic (adapted from Wang et al. [54])	-
Continuance intention	The willingness of users to continue using MMC after its initial adoption (adapted from Bhattacherjee [27])	ISCM

Note: psychological distance theory, PDT; information systems continuance model, ISCM; mobile medical consultation, MMC.

**Table 2:** Demographics of respondents (*n* = 475).

Characteristics	Frequency	Percent (%)
Gender		
Male	252	53.1%
Female	223	46.9%
Age		
20s	60	12.6%
30s	140	39.5%
40s	153	32.2%
50s	122	26.7%
Education		
High school certificate or below	57	12.0%
Technical school	94	19.8%
Undergraduate degree	267	56.2%
Master’s degree or higher	57	12.0%
Monthly household income, USD		
<700	29	6.1%
(700, 1200]	176	37.1%
(1200, 1700]	195	41.1%
>1700	75	15.8%
Year of mobile medical consultation		
Under 1 year	75	15.8%
1–2 years	224	47.2%
Over 2 years	176	37.0%
Frequency of mobile medical consultations		
Never	0	0%
Sometimes	213	44.8%
Often	262	55.2%

**Table 3:** Assessment of the reliability and validity.

Variables	Cronbach’s α	Rho_A	CR	AVE
Immediacy (IM)	0.759	0.768	0.846	0.580
Telepresence (TP)	0.735	0.737	0.834	0.557
Intimacy (IN)	0.725	0.727	0.829	0.548
Substitutability (SU)	0.754	0.758	0.844	0.576
Pandemic-induced anxiety (PA)	0.713	0.718	0.823	0.538
Satisfaction (SA)	0.726	0.726	0.829	0.549
Continuance intention (CI)	0.759	0.768	0.846	0.58

Note: composite reliability, CR; average variance extracted, AVE.

**Table 4:** Pearson product–moment correlation coefficient.

Items	1	2	3	4	5	6	7	8	9
1. Overall PD	-								
2. Immediacy (IM)	0.345 ^a^	0.761							
3. Telepresence (TP)	0.369 ^a^	0.123	0.746						
4. Intimacy (IN)	0.516 ^a^	0.399	0.341	0.740					
5. Substitutability (SU)	0.522 ^a^	0.246	0.339	0.506	0.759				
6. Pandemic-induced anxiety (PA)	0.583	0.500	0.296	0.510	0.377	0.757			
7. Satisfaction (SA)	0.670	0.407	0.398	0.559	0.523	0.418	0.733		
8. Continuance intention (CI)	0.690	0.348	0.387	0.569	0.592	0.484	0.574	0.741	
9. PDS	0.701	0.343	0.384	0.754	0.457	0.716	0.458	0.479	0.780

Note: ^a^, correlations between Overall PD subscales and total were computed with the subscale removed from the total score; the square roots of AVE are along the diagonal; correlations are below the diagonal; -: Not Applicable.

**Table 5:** Item loadings and cross-loadings.

	IM	TP	IN	SU	PA	SA	CI
IM1	0.707	0.035	0.213	0.134	0.354	0.259	0.171
IM2	0.774	0.095	0.266	0.186	0.397	0.314	0.281
IM3	0.769	0.092	0.338	0.184	0.372	0.300	0.266
IM4	0.793	0.139	0.378	0.232	0.399	0.356	0.326
TP 1	0.085	0.775	0.232	0.244	0.209	0.324	0.277
TP 2	0.072	0.740	0.254	0.285	0.239	0.284	0.321
TP 3	0.094	0.745	0.286	0.304	0.209	0.266	0.293
TP 4	0.116	0.724	0.251	0.191	0.227	0.308	0.27
IN1	0.327	0.224	0.744	0.385	0.366	0.425	0.432
IN2	0.279	0.291	0.760	0.394	0.419	0.440	0.438
IN3	0.259	0.228	0.741	0.355	0.360	0.404	0.42
IN4	0.318	0.266	0.714	0.362	0.362	0.381	0.394
SU1	0.153	0.252	0.407	0.780	0.277	0.435	0.492
SU2	0.19	0.305	0.35	0.752	0.278	0.390	0.416
SU3	0.196	0.267	0.375	0.795	0.293	0.393	0.436
SU4	0.214	0.203	0.404	0.706	0.301	0.364	0.447
PA1	0.361	0.249	0.409	0.293	0.751	0.320	0.387
PA2	0.379	0.173	0.342	0.256	0.705	0.271	0.324
PA3	0.381	0.226	0.349	0.262	0.742	0.311	0.331
PA4	0.381	0.254	0.383	0.31	0.778	0.331	0.382
PA5	0.394	0.213	0.438	0.302	0.803	0.343	0.404
SA1	0.259	0.325	0.455	0.46	0.325	0.783	0.486
SA2	0.308	0.294	0.412	0.347	0.278	0.732	0.372
SA3	0.316	0.307	0.384	0.393	0.320	0.712	0.414
SA4	0.322	0.235	0.385	0.320	0.302	0.704	0.402
CI1	0.237	0.267	0.424	0.430	0.331	0.417	0.740
CI2	0.288	0.284	0.417	0.411	0.383	0.437	0.754
CI3	0.282	0.327	0.446	0.417	0.399	0.412	0.740
CI4	0.224	0.268	0.399	0.496	0.321	0.433	0.728

Note: All items are significant under *p*-value < 0.01.

**Table 6:** Structural path analysis results.

Hypothesis	β	Standard Deviation	T Statistics	*p*-Value	Confidence Interval 97.5%	$f^{2}$	Supported
H1 IM→SA	0.246 ***	0.054	4.527	0	[0.144, 0.356]	0.078	Yes
H2 TP→SA	0.189 ***	0.045	4.199	0	[0.099, 0.280]	0.050	Yes
H3 IN→SA	0.190 ***	0.054	3.514	0	[0.081, 0.287]	0.038	Yes
H4 SU→SA	0.195 ***	0.052	3.734	0	[0.091, 0.293]	0.047	Yes
H5 SA→CI	0.456 ***	0.043	10.525	0	[0.367, 0.537]	0.293	Yes

Note 1: immediacy, IM; telepresence, TP; intimacy, IN; substitutability, SU; satisfaction, SA; continuance intention, CI. Note 2: *** *p*-value < 0.001.

**Table 7:** Moderation analysis.

Hypothesis	β	Standard Deviation	T Statistics	*p*-Value	Confidence Interval 97.5%	Supported
H6a PA × IM→SA	0.234 ***	0.045	5.176	0.000	[0.146, 0.325]	Yes
H6b PA × TP→SA	0.000	0.041	0.003	0.997	[−0.080, 0.081]	No
H6c PA × IN→SA	0.022	0.047	0.463	0.644	[−0.073, 0.107]	No
H6d PA × SU→SA	−0.154 ***	0.046	3.348	0.001	[−0.247, −0.069]	No, the effect is negative
H6e PA × SA→CI	0.088 **	0.029	3.012	0.003	[0.034, 0.149]	Yes

Note 1: immediacy, IM; telepresence, TP; intimacy, IN; substitutability, SU; satisfaction, SA; continuance intention, CI. Note 2: ** *p*-value < 0.01; *** *p*-value < 0.001.

**Table 8:** Mediation analysis.

Hypothesis	β	Standard Deviation	T Statistics	*p*-Value	Confidence Interval 97.5%	Supported
H6a IM→SA→CI	0.112 ***	0.024	4.726	0.000	[0.068, 0.161]	Yes
H6b TP→SA→CI	0.086 ***	0.023	3.728	0.000	[0.043, 0.135]	Yes
H6c IN→SA→CI	0.086 ***	0.027	3.201	0.001	[0.035, 0.141]	Yes
H6d SU→SA→CI	0.089 ***	0.027	3.278	0.001	[0.038, 0.144]	Yes
H6e PA→SA→CI	0.017	0.023	0.726	0.468	[−0.029, 0.064]	No

Note 1: immediacy, IM; telepresence, TP; intimacy, IN; substitutability, SU; satisfaction, SA; continuance intention, CI. Note 2: *** *p*-value < 0.001.

## Data Availability

Data are available upon special request from the corresponding author.

## Appendix A

**Table A1:** Questionnaire items.

Construct	Measurement Items	References
Immediacy (IM)	IM1: MMC allows me to access medical information at the best moment for me	Zhang et al. [48], Okazaki and Mendez [53]
	IM2: When I need a certain type of medical information immediately, I will use MMC	
	IM3: When I cannot wait for a certain type of medical information, I will use MMC	
	IM4: When I need to receive an urgent response, I will use MMC	
Telepresence (TP)	TP1: When using MMC, my body was in the room, but I felt my mind was inside the world created by MMC	Sun et al. [58]
	TP2: When using MMC, I felt that I was immersed in the world MMC had created	
	TP3: MMC-generated world seemed to me to be “somewhere I visited” rather than “something I saw”	
	TP4: I felt I was more in the “real world” than the “computer world” when I was using MMC	
Intimacy (IN)	INT1: When chatting with the friends using MMC, I feel like they truly understand me	Park and Lee [84], Lin et al. [64]
	INT2: When chatting with the friends using MMC, I feel like they are very close to me	
	INT3: When chatting with the patients using MMC, I feel and understand their emotion	
	INT4: Chatting with a person using MMC makes our relationship more special	
Substitutability (SU)	SUB1: MMC offers the same services as the offline medical consultation	Li [72]
	SUB2: MMC offers services in the same way as the offline medical consultation	
	SUB3: MMC satisfies the same needs as the offline medical consultation	
	SUB4: MMC is considered the same tool as the offline medical consultation for activities such as psychological counseling	
Pandemic-induced anxiety (PA)	PA1: I am anxious about the volatility of medical services prices during COVID	Wang et al. [52], Goyal et al. [85]
	PA2: I have a great fear of the supply shortage of medical resources during COVID	
	PA3: I am usually seized with panic due to the perceived phenomena of medical resources shortage during COVID-19	
	PA4: I feel dizzy or lightheaded when I read or listen to the news about COVID	
	PA5: I have trouble falling or staying asleep because I keep on thinking about the COVID issues	
Satisfaction (SA)	SA1: The MMC is trustworthy	Bhattacherjee [25]
	SA2: The MMC provider provides reliable information	
	SA3: The MMC provider keeps promises and commitments	
	SA4: The MMC provider’s behavior meets my expectations	
Continuance intention (CI)	CI1: I intend to continue using MMC rather than discontinue its use.	Bhattacherjee [25]
	CI2: My intention is to continue using MMC rather than to use any alternative means.	
	CI3: If I could, I would like to continue my use of MMC in the future	
	CI4: I intend to increase my use of MMC in the future	

**Table A2:** Common method bias measurement.

Construct	Indicator	Substantive Factor Loading (*R*1)	*R*1^2^	Method Factor Loading (*R*2)	*R*2^2^
Immediacy (IM)	IM1	0.729	0.531	0.372	0.138
	IM2	0.771	0.594	0.459	0.211
	IM3	0.778	0.605	0.461	0.213
	IM4	0.770	0.593	0.519	0.269
Telepresence (TP)	TP1	0.767	0.588	0.397	0.158
	TP2	0.747	0.558	0.416	0.173
	TP3	0.766	0.587	0.415	0.172
	TP4	0.706	0.498	0.388	0.151
Intimacy (IN)	IN1	0.740	0.548	0.587	0.345
	IN2	0.753	0.567	0.612	0.375
	IN3	0.745	0.555	0.561	0.315
	IN4	0.722	0.521	0.566	0.320
Substitutability (SU)	SU1	0.768	0.590	0.560	0.314
	SU2	0.753	0.567	0.534	0.285
	SU3	0.803	0.645	0.554	0.307
	SU4	0.710	0.504	0.537	0.288
Pandemic-induced Anxiety (PA)	PA1	0.745	0.555	0.577	0.333
	PA2	0.713	0.508	0.515	0.265
	PA3	0.746	0.557	0.544	0.296
	PA4	0.777	0.604	0.588	0.346
	PA5	0.799	0.638	0.606	0.367
Satisfaction (SA)	SA1	0.777	0.604	0.616	0.380
	SA2	0.740	0.548	0.543	0.295
	SA3	0.704	0.496	0.566	0.320
	SA4	0.712	0.507	0.534	0.285
Continuance Intention (CI)	CI1	0.751	0.564	0.576	0.332
	CI2	0.747	0.558	0.601	0.361
	CI3	0.733	0.537	0.610	0.372
	CI4	0.732	0.536	0.580	0.336
Average	N. A	0.748	0.561	0.531	0.282
